# Elective pediatric surgery: profile description of children and late referral identification

**DOI:** 10.1590/0100-6991e-20233516-en

**Published:** 2023-05-30

**Authors:** CAROLINA TALINI, ARIANA RODRIGUES DA SILVA CARVALHO, CLAUDIA SILVEIRA VIERA

**Affiliations:** 1 - Universidade Estadual do Oeste do Paraná, Programa de Pós-graduação em Biociências & Saúde - Cascavel - PR - Brasil

**Keywords:** Delivery of Health Care, Child, Elective Surgical Procedures, Surgical Procedures, Operative, Acesso aos Serviços de Saúde, Patologia Cirúrgica, Criança, Procedimentos Cirúrgicos Eletivos

## Abstract

Pediatric surgery receives great demand for referrals from primary care services in order to evaluate the need for surgical intervention. However access to this specialized evaluation and in intervention does not always occur at the appropriate time. This study aims to characterize the profile of pediatric patients electively operated in the western Paraná state region, between 2018 and 2020, and identify those who were lately referred to surgical evaluation. This is a descriptive, cross-sectional and retrospective study through the review of electronic medical records. The variables evaluated were sociodemographic data, information on underlying diseases, referral data, specialist assessment and surgical procedure. During this period, 410 patients underwent an elective surgical procedure, of which 289 were included in the research. The sample was predominantly male (72.3%) with a mean age of 57.9 months at the surgeons assessment and 59 months at the date of surgery. Most of the patients came from primary care (75%) and the most common pathology was inguinal hernia (39.1%). The mean time interval between referral through primary care and surgery was 4.98 months, and between the surgeons assessment and surgery was 1.21 months. Of the total sample, 77 (26.6%) patients were identified as being referred late for the surgical procedure. Knowing the profile of patients and the problems experienced in this region in relation to the care provided in pediatric surgery provides subsidies to propose improvement strategies not only for the health system in this location, but for several inner regions of Brazil in a similar situation.

## RESUMO

**Introdução:**

a cirurgia pediátrica representa especialidade com demanda significativa de encaminhamentos dos serviços de atenção primária para avaliação da necessidade de intervenção cirúrgica. Contudo, nem sempre o acesso a essa intervenção ocorre no momento adequado. Nesse sentido, objetivou-se caracterizar o perfil dos pacientes pediátricos operados eletivamente, no recorte temporal 2018-2020, e identificar pacientes que foram encaminhados em atraso para a avaliação com o cirurgião.

**Métodos:**

Estudo descritivo, transversal e retrospectivo mediante a revisão de prontuários eletrônicos. As variáveis avaliadas foram dados sociodemograficos, informações das doenças de base, dados do encaminhamento, da avaliação do especialista e do procedimento cirúrgico.

**Resultados:**

neste período 410 pacientes foram submetidos a procedimento cirúrgico eletivo, dos quais 289 foram incluídos na pesquisa. O sexo masculino (72,3%%) foi predominante, com idade média de 57,9 meses na avaliação pelo cirurgião e 59 meses na data da cirurgia. A procedência dos pacientes foi na maioria da atenção básica (75%) e a patologia mais incidente foi a hérnia inguinal (39,1%). O intervalo de tempo médio entre o encaminhamento pela atenção primária até a realização da cirurgia foi 4,98 meses e entre a avaliação pelo cirurgião e a realização da cirurgia foi 1,21 meses. Do total, 77 (26,6%) pacientes foram identificados como encaminhados em atraso para a realização do procedimento cirúrgico.

**Conclusão:**

conhecer o perfil dos pacientes e os problemas vivenciados nesta região em relação aos atendimentos prestados na cirurgia pediátrica apresenta subsídios para propor estratégias de melhoria não só do sistema de saúde local, mas de diversas regiões interioranas do Brasil em situação semelhante. .

## INTRODUCTION

Pediatric surgery represents a significant demand for referrals from primary care services to assess the need for surgical intervention. There are several conditions that can be surgically corrected on an outpatient basis, in a day hospital, such as phimosis, inguinal hernias, umbilical hernia, hydrocele, and cryptorchidism. According to De Jesus et al. (2009), the estimated demand for surgical care in the Brazilian pediatric population is approximately 10% of children.

However, among the distribution of medical specialties in the country, the specialty of pediatric surgery has a low number in all regions, not reaching one doctor per 100,000 inhabitants in any. Most of the workforce is concentrated in the Southeast and South regions of the country, especially in large cities^1^. Limitations are thus identified in serving the population of other regions and in cities in the interior, even more evident when considering the great distances involved in accessing health services, the social and labor problems of economically less favored families, the precariousness of transport systems in various regions, restrictions on social assistance, and practical difficulties in the interaction between services^2^.

This heterogeneous distribution of professionals is determined by market availability, level of remuneration, structuring of support services, and quality of life offered to professionals^3^ ^,^ ^4^. The scarcity of professionals associated with the unequal distribution in the country makes it difficult to reach the specialist, often delaying indication and surgical correction^1^. In addition, due to the lack of knowledge of the appropriate age for surgical interventions, many children are only evaluated by the specialist in a later period^5^.

Besides the small number of specialists in pediatric surgery, there are also issues specific to the health care model and the organization of health care actions and services in the Brazilian Public Unified Health System (SUS)^6^. The levels of care that make up the health services network must act in an articulated way, developing actions to guarantee the completeness of care to the user. In this context, access to specialized care is one of the greatest difficulties of SUS, due both to the insufficient supply of diagnostic and therapeutic actions and to the modes of organization and functioning of specialized care, including, for example, the quality and quantity of referrals and requests, which generates an important overload in this care segment. Principles and guidelines should thus be considered, such as equity, transparency, timely access to services and their adequate use, based on the needs of each user, aiming at comprehensive care^7^.

One of the great challenges of primary care is deciding when to refer the patient to a specialist in secondary or tertiary care8. Regulatory action must be based on referral protocols, ordering instruments for the different levels of care complexity and on clinical protocols, which deal with the form of intervention for pathologies, supporting therapeutic decisions^7^. In order for the Primary Health Care (PHC) service to be able to organize the flow of care and referrals to specialized care for the population assigned to its territory, it is necessary to know the profile of these populations. To this end, profiling children undergoing pediatric surgery will contribute to proposing measures aimed at reducing the delay in referrals of potentially surgical patients to be evaluated by a specialist. Furthermore, performing the surgeries within the expected age range will provide positive results in the long run, both in terms of reducing complications resulting from the delay in performing the necessary surgical correction, and in reducing family stress. This study aims to characterize the profile of pediatric patients electively operated in the Western region of Paraná, as well as to identify patients who were referred late for evaluation with the surgeon.

## METHODS

We carried out a quantitative, descriptive, cross-sectional, and retrospective study, based on secondary data from three different outpatient clinics linked to SUS, in the West region of the Brazilian State of Paraná, belonging to the 10th Health Region.

We collected data from the electronic medical records of patients treated by the specialty of Pediatric Surgery, using the name of the patients as a filter. We also collected information from the primary care physician’s referral brought by the patients to the consultation and recorded in the outpatient medical records, as well as in the hospital medical records made at the time of surgery. From the review of the medical records of the assisted children, we could identify the age at which the procedure was performed, assessing whether there was a delay in referrals.

The time frame of the study was from September 2018 to March 2020, chosen because it represents the period between the start of pediatric elective surgeries in the SUS in the 10^th^ Health Region of Paraná and the time when elective surgeries were stopped for the first time and had a great impact on their performance due to the COVID-19 pandemic.

The study involved data from the entire population of children undergoing an elective surgical procedure, aged 0 to 13 years old, seen during the study period in the PHC, by pediatricians or not, and who were referred for evaluation by a specialist. We excluded data from patients who had incomplete medical records or those records that were not found due to changes in the spelling of the name or incomplete surnames.

The variables collected and analyzed were child demographics, epidemiological data, underlying pathology, information regarding the referral by the primary care unit, data about the evaluation by the specialist, and data about the referral and performance of the surgical procedure.

We tabulated the collected data in Microsoft Excel 2010 and analyzed them in the SPSS version 26.0 statistical software, using descriptive statistics (mean, standard deviation, minimum and maximum, mode) to characterize the profile of pediatric patients electively operated on during the study period. We tested the variables for normality (Shapiro-Wilk test) and homoscedasticity (Levene test). Descriptive analyzes were performed for all variables, using percentage proportion measures for categorical variables, and central tendency and dispersion for quantitative ones, considering possible missing data.

We used the chi-square test to analyze the relationship between patients subject to late referral, postoperative complications, postoperative evolution (with or without recurrence), and to compare postoperative evolution with postoperative complications. We used analysis of variance (ANOVA) to study the distribution of pathologies according to the ages at which the children were in the referral assessment, in diagnosis, in the evaluation by the surgeon, and in the preoperative consultation and surgery. To verify the difference between the groups that were considered statistically significant, when comparing age (at different times) and the group of pathologies presented by the children who composed the study sample, we used the Tukey’s post hoc test. We considered p-values <0.05 as statistically significant.

The study complied with all the regulations of Resolution 466/2012, approved by the institution’s Ethics and Research Committee, Opinion 5,023,889 (CAAE:50835621.1.0000.0107).

## RESULTS

During the analyzed period, 410 pediatric patients were operated on an elective basis, of whom 121 were excluded from the sample according to the exclusion criteria. Thus, data from 289 patients comprised the study sample, that is, 70.4% of the patients operated in the period.

Regarding the sociodemographic characteristics of the sample, we observed in (Table 1) that 209 (72.3%) patients were male and 152 (52.6%) were residents of the main city of the Health Region. Thirty (10%) patients had other comorbidities, neurological conditions being the most frequent, in eight (26.7%) children.

**Table 1:** Characteristics of the study sample (n=289).

Variable	n (%)
Sex	
Male	209 (72.3)
Female	80 (27.7)
City of origin	
Main Health Region city	152 (52.6)
Other municipalities in the Health Region	137 (47.4)
Comorbidities	
No	259 (89.6)
Yes	30 (10.4)
Variable	n (%)
Neurological conditions	8 (26.7)
Respiratory conditions	6 (20)
Mental conditions	3 (10)
Gastrointestinal conditions	3 (10)
Hematological conditions	1 (3.3)
Dermatological conditions	1 (3.3)
Prematurity	1 (3.3)
Obesity	1 (3.3)
More than one health condition	6 (20)

*Source: Survey data.*

As for type of surgery, the most common was inguinal herniorrhaphy (n=113; 39.1%), followed by postectomy (n=47; 16.3%), umbilical herniorrhaphy (n=44; 15, 2%), and orchidopexy (n=27; 9.3%) (Table 2).

**Table 2:** Types of pediatric surgeries performed from 2018 to 2020 (n=289).

Surgeries	n (%)
Inguinal herniorrhaphy	113 (39.1)
Postectomy	47 (16.3)
Umbilical herniorrhaphy	44 (15.2)
Orchidopexy	27 (9.3)
Epigastric herniorrhaphy	10 (3.5)
Exeresis of skin lesion	10 (3.5)
Surgical repair of hydrocele	9 (3.1)
Branchial cyst excision	7 (2.4)
Sublingual frenotomy	4 (1.4)
Branchial fistula excision	2 (0.7)
Exeresis of cyst on browbone	2 (0.7)
Thyroglossal cyst excision	2 (0.7)
Correction of labia minora synechiae	2 (0.7)
Exeresis of non-articulating polydactyly	1 (0.3)
Cervical lymphadenectomy	1 (0.3)
More than one concurrent procedure	8 (2.8)

*Source: Survey data.*

We assessed the age of operated patients at different times. Initially, we observed that patients’ average age when referred for evaluation by the surgeon was 61 months. The average age of patients at diagnosis of the pathology by the physician who provided the initial care and who led to the referral to the specialist was 58 months. Furthermore, the mean age at the specialist’s assessment was 57 months and on the date of the surgery, 59 months.

We also assessed age separately by pathology, considering the most frequent ones (Table 3). The comparison between the children who needed to undergo surgery due to phimosis and those who underwent inguinal herniorrhaphy showed a statistically significant difference when analyzing age at the preoperative consultation, specialist assessment, and age at the time of surgery (p<0.001 for each).

**Table 3:** Children’s age by pathology (in months), from referral to surgery.

Age	Pathology	Mean ± SD	p**
Age at specialist assessment (n=281) p*=0.002	Inguinal hernia (n=113)	47.5 ± 40.1	
	Umbilical hernia (n=44)	60.1 ± 31.5	>0.05
	Phimosis (n=47)	75.1 ± 38.0	<0.001
	Hydrocele (n=9)	48.0 ± 26.2	p>0.05
	Others (n=68)	62.9 ± 47.4	p>0.05
Age at referral (n=57) p*=0.704	Inguinal hernia (n=17)	53.2 ± 38.0	
	Umbilical hernia (n=14)	43.0 ± 26.3	
	Phimosis (n=11)	70.0± 36.2	
	Hydrocele (n=2)	56.0 ± 2.8	
	Others (n=23)	67.6 ± 53.7	p>0.05
Age at diagnosis (n=56) p*=0.783	Inguinal hernia (n=18)	51.4 ± 37.9	
	Umbilical hernia (n=4)	43.0 ± 26.3	
	Phimosis (n=11)	63.4 ± 28.3	
	Hydrocele (n=2)	56.5 ± 2.1	
	Others (n=21)	65.7 ± 54.8	p>0.05
Age at preoperative consultation (n=281) p*=0.002	Inguinal hernia (n=113)	47.5 ± 40.2	
	Umbilical hernia (n=44)	60.1 ± 31.4	p>0.05
	Phimosis (n=47)	75.2 ± 37.9	<0.001
	Hydrocele (n=9)	48.0 ± 26.2	p>0.05
	Others (n=68)	62.3 ± 47.8	p>0.05
Age at surgery (n=281) p*=0.002	Inguinal hernia (n=113)	48.7 ± 39.9	p>0.05
	Umbilical hernia (n=44)	61.1 ± 31.3	p>0.05
	Phimosis (n=47)	76.0 ± 37.9	<0.001
	Hydrocele (n=9)	49.0 ± 26.2	p>0.05
	Others (n=68)	64.3 ± 46.9	p>0.05

Source: Research Data. SD: Standard Deviation; p*: one-way ANOVA; p**: Tukey’s post hoc test.

As for the place of referral, we found that 42 (75%) patients came from PHC units, while 13 of them (25%) were referred from the hospital or by doctors from the specialty clinic itself. The diagnosis of the pathology that motivated the referral was mostly made by the physician at the PCH unit (80.3%). A total of 44 (15.7%) patients had already been previously referred for evaluation by a surgeon, and 10 (3.5%) had been evaluated in pediatric surgery services in other reference municipalities in the state, and of these, two (0.7%) had surgery indicated, however, none of them underwent the procedure.

Regarding to complementary exams, we observed that 40 (32%) patients arrived for the evaluation with the specialist carrying previously performed imaging exams and seven (2.4%) had undergone the imaging exam requested by the surgeon, that is, after the first consultation with the specialist.

When evaluating the time to perform the surgery, we found that 24 (8.3%) patients took more than 60 days to be operated after the surgery guide was issued, the others having been operated before that time. Most of the children, 187 (64.7%), were operated on 30 days after the surgeon’s assessment. The most frequent reason for waiting for surgery for more than 60 days was the surgical queue at the hospital, in 15 (5.2%) cases.

We also evaluated the time interval between the referral of the patient by the primary health care and surgery, this wait ranging between 0.5 and 17 months, with 11 (21.2%) patients waiting for two months, nine (17.3) waiting for one month, and eight (15.4) patients waiting for four months. The mean time interval between referral through primary care and surgery was 4.98 months, with a median of four months, and the mean interval between the surgeon’s assessment and surgery was 1.21 months, with a median of one month (Table 4).

**Table 4:** Interval (in months) from referral to surgery (n=289).

Variables	n (%)	Mean ± SD	Median	Range
Interval between referral by PHC and surgery	52	4.98±4.26	4	0.5 17
Interval between specialist evaluation and surgery	289	1.21±0.92	1	0 8

*Source: Survey data.*

Of the total, 77 (26.6%) patients were identified as being referred late for the surgical procedure, and among them, we could not find the reason for the delay in the medical records of 74 (25.6%). Late referral happened for 54 out of the 113 (47.7%) children with inguinal hernia, three out of the nine (33.3%) patients with hydrocele, and 17 out of the 27 (62.9%) patients with cryptorchidism. The comparison of delay by pathology showed statistical significance (p<0.01).

When evaluating any findings during surgery that could be compatible with this delay in patients’s referral, we observed that, in 14 (7.3%) of them, the testicle was small in relation to that expected for their age, and in one (0.5%), the testis was absent. According to the surgical findings, the hypothesis of risk of testicular atrophy and infertility as a future implication was present in 15 (7.8%) patients. Of the operated patients, 128 (68.1%) did not present alterations that could be related to the delay in surgery at the time of the evaluation.

The mean postoperative follow-up time was 2.66 ± 2.82 months, ranging from one to 18 months. During the follow-up period, five (1.7%) had surgery-related complications, namely surgical wound infections, skin suture dehiscence, suture granuloma, and large seroma. Of the patients who had postoperative complications, two (40%) were referred for delayed evaluation and three (60%) were referred at the appropriate time, with no statistically significant difference (p=0.49). The five cases of recurrence of the underlying pathology were patients referred for delayed evaluation, with no statistical difference (p=0.20). None of the patients who had postoperative complications had concomitant recurrence of the underlying pathology (p=0.75).

Of the operated patients, five (2.4%) had recurrence of the underlying pathology, two of which had phimosis, one had thyroglossal cyst, one had inguinal hernia, and one had umbilical hernia. Of the total sample, 208 (72%) children were discharged from the outpatient clinic, 80 (27.7%) of them did not return for the postoperative consultation, and one (0.3%) returned only for the first consultation and was not followed afterwards.

## DISCUSSION

Elective surgeries occur when doctors and users schedule the surgical event to be performed in advance, when there is no emergency. These procedures are performed in the hospital and in outpatient settings and, in the period 2015-2018, 1,431,928 outpatient elective surgeries and 798,610 hospital surgeries were performed in the state of Paraná. During the evaluated period, there was an increase of 34% in outpatient elective surgeries and 22% in hospital elective surgeries. Despite the growing number of elective surgeries performed, this action is still identified as a repressed demand in all Health Regions of the state. Given this demand and the difficulty in supply, there is a need to organize and expand access to elective surgeries, with the implementation of protocols and qualification of queues^9^.

The characterization of the pediatric surgical patients treated at the Health Region in question within the time frame stipulated for the study showed that 72.3% of the patients were male, 52.6% from the main city in the Health Region, and the most frequent pathology was inguinal hernia (39.1% of cases). Corroborating what is described in the literature, the main elective surgeries that are commonly performed on an outpatient basis in pediatric patients in this sample consisted of inguinal herniorrhaphy, followed by postectomy, umbilical herniorrhaphy, and orchidopexy. According to previous reports in the literature, abdominal wall hernias (inguinal, umbilical, and epigastric) are the causes of 34% of referrals for surgical evaluation, with a mean age of four years at referral^5^ ^,^ ^10^. Most conditions with the possibility of outpatient surgical correction affect males in a greater proportion or exclusively^10^. Therefore, the prevalence of boys is higher in investigations involving pediatric pathologies, data confirmed in this study.

Inguinal hernias are one of the most common surgical conditions in childhood and occur in approximately 5% of newborns, with an increased incidence of 11% in premature infants^11^. The most important reason for early surgical repair of inguinal hernias is the increased risk of incarceration, especially during the first year of life, with an estimated risk of 30%, and among patients who have incarceration, there is a risk that 30% of them will evolve with testicular atrophy^12^. Incarceration and complication rates increase with the longer waiting time for the surgical procedure^13^, complications that we did not observe in our series.

Among the most common surgical pathologies, the one rendering more discussion about the appropriate moment for surgical repair is cryptorchidism. It is one of the most common congenital disorders in males, with an incidence of 4% of full-term boys and up to 30% of premature babies^14^. Despite the possibility of spontaneous descent of the testicle into the scrotum after birth, its occurrence proved to be uncommon after three months of life^15^ and rare after six months^14^. The age at which the surgical intervention is performed is an important predictive factor for the future fertility of these patients. Furthermore, patients with cryptorchidism have an increased risk of developing testicular tumors, especially those treated surgically after puberty^16^. Over the past five decades, the age indicated for surgical intervention has changed significantly. In 1986, the recommendation of the American Academy of Pediatrics was for surgery between 48 and 72 months. With the completion of studies that demonstrated a reduction in the number of germ cells below normal levels at 12 to 24 months of age, the recommendation was changed to surgery at 12 months of age in 1996^14^. Considering the low probability of spontaneous testicular descent after three months of life, the British Association of Pediatric Urologists recommended reducing the surgical age to six to 12 months from 2011 onwards, suggesting a benefit in earlier surgical intervention, at three months of life^14^. In this study, the average age at referral of patients with cryptorchidism for evaluation by the specialist was 71.5 months, and in the evaluation by the surgeon, 56.4 months, averages well above what is considered ideal for the treatment of this pathology.

We investigated whether patients with cryptorchidism already had any findings during surgery that could be compatible with the delay in referral, and we found that in 7.3% the testicle was small in relation to what was expected for their age. According to the findings, the hypothesis of risk of testicular atrophy and infertility as a future implication was present in 7.8% of cases, however there was no previous measurement of testicular size to characterize objective values for this subjectively observed reduction.

According to current consensus, surgery should be performed not after 18 months of age^15^. In our sample, the mean age at the time of orchidopexy was 58.3 ± 54.9 months. A recent population cohort study concluded that for every six months of delay in performing orchidopexy, there is a 6% increase in the risk of developing cancer, a 5% increase in the need to use technologies for assisted reproduction, and a 1% reduction in the rates of paternity^17^. Even in developed countries, such as Sweden, a minority of boys are operated on during this period^18^, especially due to the large volume of patients in the few pediatric care centers. Many patients in this sample were operated on at a much later stage than expected, some cases surpassing the age of eight for its performance, which, according to the demonstrated literature, has a very negative impact on the future development of the boys’ testicles. This could be avoided if referrals and evaluation with the specialist were carried out early, as indicated in the literature.

The mean age at the specialist’s assessment was 57 months and the mean age at the time of surgery was 59 months. Of the total, 64.7% underwent surgery 30 days after the surgeon’s evaluation. The mean time interval between referral through primary care and surgery was 4.98 months, and the mean interval between evaluation by the surgeon and surgery was 1.21 months. Among the 289 patients of the study sample, 77 (26.6%) were identified as being referred late, with delay being more frequent in patients with cryptorchidism, in 62.9%.

The waiting time for elective surgery is a relevant issue in accessing health care. A wait of more than three months is considered excessive in several countries in the Organization for Economic Co-operation and Development (OECD) group^19^. The countries with the shortest waiting times for elective surgery are also those with the highest percentages of outpatient surgeries. A study carried out in Portugal showed a reduction in the waiting list for elective surgeries from six to eight months to values between 2.5 and 3.5 months, also verifying an increase in the percentage of outpatient surgeries performed in the same period19. Previous studies have already shown an average waiting time of 31 days in the public health system, from the appointment with the specialist to the surgical procedure20 and a time that never exceeded two weeks in this interval^21^. In the present study, even with surgeries being performed on an outpatient basis, the waiting time between the surgeon’s assessment and the procedure was on average 1.21 months, and the time from referral to surgery was 4.92 months.

The diagnosis of a surgical pathology in itself is already a stress factor for the pediatric patient, as well as for their family members, and the long wait for treatment and resolution of the condition tends to be an aggravating aspect in this scenario. In addition to the negative psychological and social effects caused by this wait, there is also the negative economic effect arising from the need for additional interventions and hospitalizations due to the underlying pathology. Furthermore, the consequences of the delay in performing the surgery and the result obtained with the treatment may be impaired due to the delay in the procedure^22^. Around five billion people throughout the world, including children and adults, do not have safe access to surgical and anesthetic procedures when needed^23^.

Many factors are known to be associated with precarious care for children, especially in underdeveloped countries, such as poor transport conditions, distances between large centers and small towns in the interior, poverty, lack of knowledge of those responsible for the children, cultural beliefs, impossibility of funding of health procedures by families, limitation in the number and location of specialized care centers, limitation in the availability of specialists, difficulties in carrying out complementary exams, and low availability of safe pediatric anesthesia^23^. Even in developed countries such as Canada, it has recently been shown that compliance rates regarding waiting times for surgical procedures in pediatrics are far from ideal and that additional efforts are needed to improve the proportion of patients seen in reference targets^22^.

This study has several limitations, among which we can mention the data collected retrospectively (secondary data), the information from a single health regional in a single state of Brazil, the fact that not all medical records were complete, especially in relation to the referral information and medical data prior to the evaluation with the specialist, as well as data related to the loss of postoperative follow-up of several patients, which may have occurred due to the COVID-19 pandemic and the impairment of outpatient care during this period. However, it raises important points to be further studied and debated in the area of pediatric surgery in this country.

Possible improvements to the found scenario include training of physicians who aid in primary care, bearing in mind that these are pathologies that are not always generally known to everyone, and issues such as appropriate ages for referral to a specialist may go unnoticed by these professionals, in addition to the establishment of new referral flows and prioritization of patients with pathologies in which the delay in surgery has greater future implications for patients.

## CONCLUSION

The characterization of pediatric surgical patients showed that the majority were male and that the most frequently identified pathology was inguinal hernia. After epidemiological characterization of the study participants regarding referral, specialist assessment, and surgical procedure, 26.6% of patients were referred late for surgery. The pathology most frequently associated with delay in referral, evaluation by a specialist, and surgery was cryptorchidism, a condition whose treatment delay has an important negative impact in the future.

We suggest that the shortage of professionals in the specialty and lack of coordination in the primary care network, which makes articulation between the network services difficult, can be raised as contributing factors to the difficulties encountered in this process between referral and surgery.
